# Development and evaluation of image-series for portion size estimation in dietary assessment among adults

**DOI:** 10.1017/jns.2020.58

**Published:** 2021-01-13

**Authors:** Lorentz Salvesen, Dagrun Engeset, Nina C. Øverby, Anine C. Medin

**Affiliations:** Department of Nutrition and Public Health, Faculty of Health and Sport Sciences, University of Agder, PO Box 422, Kristiansand 4604, Norway

**Keywords:** Dietary assessment, Validation, Methodology, Portion size estimation, Images

## Abstract

Portion size images are advantageous in dietary assessment. The aim of the present study was to develop and validate new culturally specific image-series for portion size estimation to be used in a new Norwegian version of a British web-based dietary assessment tool (myfood24). Twenty-three image-series of different foods, each containing seven portion size images, were created and validated in a group of adults (*n* 41, 58 % female) aged 19–44 (median 23), out of which 63 % had higher (tertiary) education. The participants compared 46 portions of pre-weighed foods to the portion size images (1886 comparisons in total). Portion size estimations were either classified as correct, adjacent or misclassified. The weight discrepancy in percentage between the chosen and the correct portion size image was also calculated. Mann–Whitney *U* tests were used to explore if portion size estimation accuracy differed across sample characteristics, or if it depended on how the foods were presented. For thirty-eight of the forty-six presented food items, the participants selected the correct or adjacent portion size image 98 % on average. The remaining eight food items were on average misclassified by 27 % of the participants. Overall, a mean weight discrepancy of 2⋅5 % was observed between the chosen and the correct portion size images. Females estimated portion size more accurately than males (*P* = 0⋅019). No other significant differences in estimation accuracy were observed. In conclusion, the new image-series performed satisfactorily, except for the image-series depicting bread, caviar spread and marzipan cake, which will be altered. The present study demonstrates the importance of validating portion size estimation tools.

## Introduction

Improving diet quality could potentially prevent one in five deaths globally^(1)^. In Norway, diet is one of the modifiable risk factors that cause the most deaths, along with high blood pressure and smoking^(2)^. Clearly, there is a need for effective strategies to improve diet, which depend on accurate data on dietary intakes which require valid dietary assessment methods. Portion size estimates is a critical element in dietary assessment^(3–6)^. A recent systematic review by Amoutzopoulos *et al.* shows that there is a lack of validated portion size estimation tools, and consequently a pressing need for more validation studies^(7)^.

Self-administered web-based dietary assessment methods represent a favourable option to the standard methods of assessing diet by paper or telephone. They are readily available for the participant at any time and place, and reduces the cost of conducting a dietary assessment and the burden for both the participants and the researchers^(8,9)^.

An example of a digital version of the traditional 24-h recall method, is the self-administered web-based 24-h recall system myfood24, developed in Leeds, England^(10)^. The myfood24-system has been validated in various settings^(11–13)^ and adapted to several country-specific versions (Australia, Denmark and Germany^(14)^). Norway lacks a self-administered web-based 24-h-recall-system for adults. Hence, we have recently adapted the British myfood24 for Norway, including the image-series for portion size estimations.

In terms of validity, the web-based dietary assessment tools and standard methods have been shown to be in close agreement^(13,15)^. Along with the development of web-based dietary assessment tools over the last years, the traditional portion size estimation tools, e.g. food models and household measures, have been largely replaced by digital images of portion sizes. Importantly, food images have been shown to be more accurate compared with food models and household utensils in the review by Amoutzopoulos *et al.*^(7)^ Further, two studies included in their review compared digital images and printed images, and reported no statistical difference in accuracy between these image types^(16,17)^. It is established that the number of images in an image-series affects the portion size estimation accuracy, favouring a high number of images^(18,19)^, which is more convenient when using a digital format *v.* printed images.

Reporting accuracy in dietary assessment is affected by a number of factors, such as demographics (sex, age, education and body mass index) and the dietary assessment method used. Previous research shows conflicting results regarding estimation accuracy between the sexes when using photographs or images to estimate portion size, where some found greater underestimation among male participants^(18,20)^, while others found no statistical difference^(21,22)^. Moreover, previous studies have not observed associations between the educational level and the perception of portion size^(20,21)^.

The overall aim of the present study was to develop new culturally specific image-series for portion size estimation and assess their validity. As part of validating the image-series, we explored whether portion size estimation accuracy differed by sex, level of education, and whether participants had studied food science or nutrition. Furthermore, we explored whether presenting the food items differently in relation to how the food was depicted in the image-series would affect how accurately the participants estimated the portion sizes.

## Methods

The method section is divided into three parts: part one describes the myfood24-system; part two describes the development of image-series to aid portion size estimation in a Norwegian version of myfood24; part three describes the design of the validation study in which the new food portion image-series were assessed.

### The myfood24-system

The dietary assessment tool myfood24, short for ‘Measure Your Food On One Day’, is a web-based 24-h recall system^(10)^. The system is self-administered by the participant and is structured around pre-defined meals, and includes features such as searching the available database for food items, aids for portion size estimations with images and a recipe-builder.

In adapting myfood24 to a Norwegian setting, a food composition database tailored for the Norwegian population was compiled using the Norwegian Food Composition Database 2019^(23)^ supplemented with food composition data for missing traditional Norwegian dishes from other sources. Portion size images for the Norwegian version were tailored to a Norwegian food culture.

### Development of new food portion image-series for myfood24 for Norway

#### Deciding what food items to depict

The need to add new image-series for typical and frequently eaten Norwegian food to the Norwegian version of myfood24 was identified after a preliminary examination of the fifty-nine image-series from the British myfood24.

The choice of which foods to develop image-series for in this study was guided by the selection of image-series used in the national dietary survey, Norkost 3, conducted among adults in Norway^(24)^. In addition, first-hand experiences using these image-series in previous studies among adults^(25)^, and the portion size photo booklet ‘Matmallen’^(26)^, a meal model tool developed by the Swedish National Food Administration, were looked at.

All food items selected to be included in the new image-series had to be listed in the Norwegian food composition table. Each image-series’ potential to be used for portion size estimation for more than one food item (a proxy for similar foods) were considered, favouring those suitable as a proxy for image-series development (e.g. muesli being a proxy for different types of breakfast cereals).

Dishes that may vary largely regarding the ingredient list (e.g. for tacos) were regarded as unsuitable to be included in the image-series.

#### Deciding the sizes of each portion

A high number of portion size images has shown to provide more accurate portion size estimation; for example, eight images presented simultaneously is shown to be more accurate compared to a single ‘average’ image^(18)^ or four images^(19)^ in previous studies. Hence, the newly developed image-series included seven images with increasing portion sizes, in line with the existing British image-series^(27)^.

Four different criteria were used during the development of the portion sizes for the image-series. First, one of the three middle portion sizes (images 3–5) should be an approximate of the Norwegian or Swedish standard serving^(28–30)^. Second, the portion size extremes were selected based on experiences as nutritionists and what was considered a plausible portion size. Third, when applicable, the food packaging or food container was taken into account (e.g. a can of beans). Finally, fixed percentage weight increments were applied to the image-series, in line with the image-series in the British myfood24 (e.g. for blueberries, we applied a fixed percentage weight increment of 50 %, resulting in the following portion sizes for blueberries in grams: 22, 33, 50, 74, 112, 168, 251). The rationale for using a fixed percentage increment is that it makes the difference between portion sizes clearly visible. In the present study, a fixed percentage increment was used for all but one of the image-series. The exception was the image-series for bread, in which a fixed increment in gram was used, to apply the Norwegian standard servings for a thin, medium and thick slice of bread as the middle portion size images (images 3–5)^(28)^.

#### Photographing and editing the image-series

**Fig. 1.**
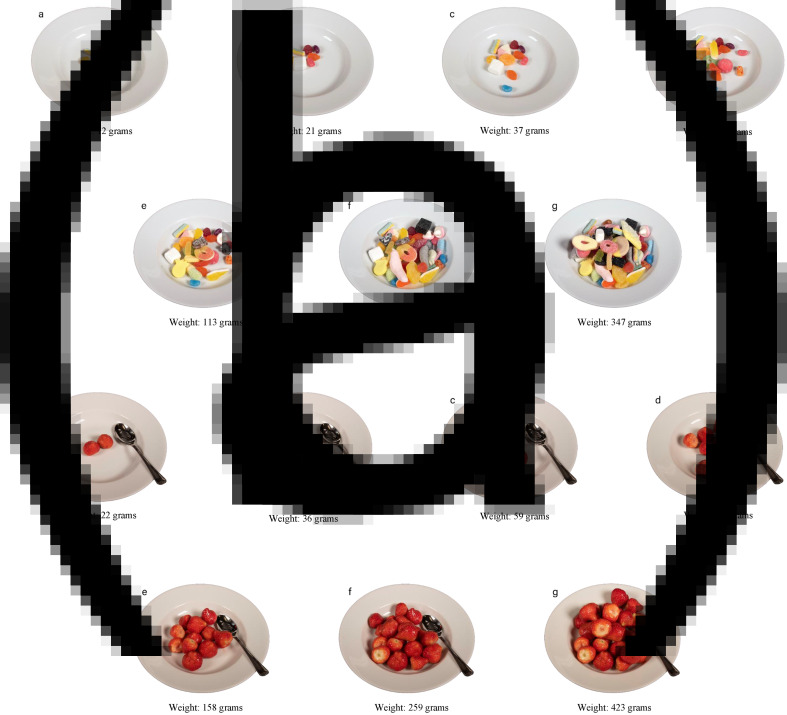
Examples of image-series with the letters A–G edited in to assist portion size image identification: (a) candy and (b) strawberry.

An in-house professional photographer at the University of Agder (UiA) was engaged to photograph the food and create the image-series (Supplementary File S1, Table S1 and Supplementary File S2, Figs. S1 and S2). Photographing was done in two separate sessions. Food items that needed cooking or preparations (e.g. mince and stews) were prepared immediately before photographing. Two identical kitchen weights with 1-g increments (Swordfish SFKSW14E) were used to ensure correct weight. Food items were placed naturally on the plate, as done in real life, meaning that they were not arranged in an aesthetical manner. During the post-processing of the images, the background for each image-series was made transparent. To assist participants in identifying the different portion size images during the study, the letters A–G were embedded next to the food items on each image in the image-series (Fig. 1). In total, twenty-three image-series were developed (image-series not presented in the paper are available in Supplementary File S3, Figs. S1–S18).

### Validation of portion size image-series for the Norwegian version of myfood24

#### Design of the validation study

The perception approach was applied to evaluate the image-series^(18)^. This entails presenting participants with pre-weighed food items in real time and having them estimate the portion sizes using the amounts depicted in images. This approach does not require the need for participants to conceptualise or rely on their memory.

The validation study was performed at four different time points, all in one day. The location was a large training kitchen at the university campus at UiA, in South of Norway. Each of the foods or dishes depicted in the twenty-three newly developed image-series were presented twice during the validation study, of which ten were presented with a different plate or bowl than depicted. The participants estimated the forty-six portion sizes in real time by observing the presented food items and choosing the portion size image they perceived as the same quantity. Participants were instructed not to discuss the portion size estimations with the other participants, nor to taste or eat the presented foods.

**Fig. 2.**
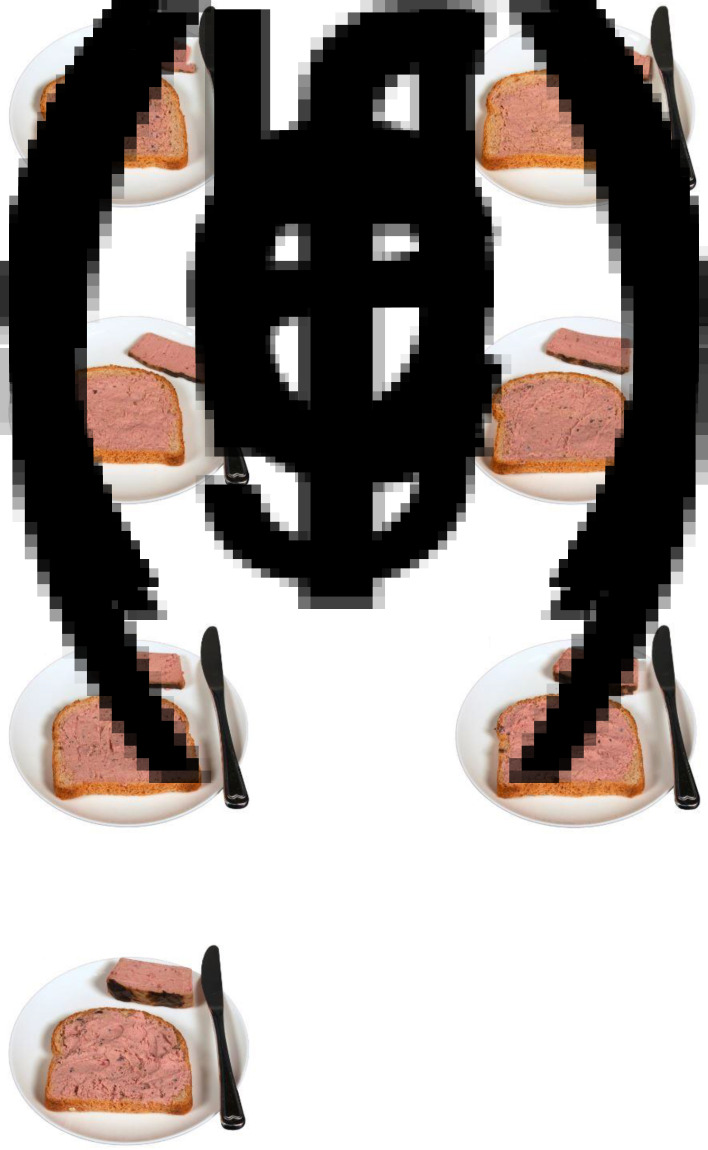
Example of an image-series used to estimate portion size in the digital questionnaire for ‘Dish 21. Liver-pâté’.

A digital questionnaire was developed in SurveyXact^(31)^ to be used on handheld computers (Chromebook) or tablets (iPad) during the data collection. The questionnaire displayed the image-series corresponding to the presented food items as forty-six separate questions (example in Fig. 2). Portion size image weight was not shown. Successively, participants were asked questions covering demographic information: sex, age, level of education, and whether the participant had studied food science or nutrition.

#### Recruitment

Forty-one participants were recruited at the university campus, using convenience sampling over a period of 14 d. A variety of different approaches was used in the recruitment phase, including social media and personal networks. Posters and flyers were placed and handed out on strategic places throughout the university campus (e.g. in the cafeteria, bulletin boards and classrooms). Recruitment was also carried out in lectures and among students, colleagues and employees at the university.

To be included in the study individuals had to be between the age of 18–45, speak Norwegian and be willing to be present at the university campus at one of four scheduled times. Individuals who had recorded their diet (e.g. using a food diary) during the last year were excluded from the study.

Participation in the study was voluntary. Written informed consent was obtained from all subjects. A gift card at a coffee shop, valued at 66 NOK (equivalent to approx. 7 Euros), was used as an incentive to recruit participants. Participants did not provide any person-identifying information during the study. All procedures involving research study participants were approved by the Norwegian Centre for Research Data (reference number: 637822) and the ethics committee of the Faculty for Health and Sport Sciences, University of Agder.

#### Preparations for the validation study

Each of the twenty-three food items depicted in the image-series were presented twice in the kitchen facility; hence, a total of forty-six servings of foods were presented for the participants in the present study. Each food item was presented once with identical weight relative to a portion size image, and once with an altered weight of 25 % of the differential to the adjacent portion size (e.g. two servings of brownies were presented: one weighing the exact same as a corresponding portion size image weight of 134 g, and the other weighed 148 g). The foods presented were numbered as an aid for the participants. The presentation of the portion sizes was randomised as follows: 50 % of the food items were presented as a middle portion size (images 3–5); 25 % as a smaller portion size (images 1–2) and 25 % as a larger portion size (images 6–7). Fourteen food items were presented with an increased weight relative to depicted. However, none of the presented portion sizes was smaller than image 1 or larger than image 7.

Food items that needed cooking were prepared the day before and stored in a cold storage room. Foods that could stay overnight were weighed and prepared the day before the study (e.g. candy and muesli). The same kitchen weights as used during the development of the image-series were used to ensure correct portion sizes (Swordfish SFKSW14E). All remaining dishes and spreads were weighed and prepared the morning of the study, but spreads were replaced before the last study round for visual reasons. Foods were plastic-covered and refrigerated between study rounds. Ten food items were presented with a different plate or bowl than depicted in the image-series to examine the effect of presentation method on estimation accuracy. No cutlery was presented with the food items.

#### Statistics

Descriptive analyses were conducted to explore the participants’ characteristics. Sex, level of education and participants having studied food science or nutrition are presented as frequency and percentage. Participants’ age and years of studying food science or nutrition are presented as median (25 %, 75 %), as these data were not considered as normally distributed.

Participants’ estimates of portion sizes assisted by the image-series were classified into correctly classified, adjacent, lightly misclassified or grossly misclassified. A portion size estimate classified as correct is defined as a perfect match between the portion size image chosen by the participant and the portion size of that same food or dish presented to the participant. A portion size estimate classified as adjacent is defined as a partially match, that is, when the participant selected the portion size image closest to the image corresponding to the presented portion size. A lightly misclassified estimate is defined as a partially mismatch, that is, when the participant chose an image of a portion size situated 2–3 images distant from the correct portion size image for the presented serving of food. A grossly misclassified estimate is defined as a complete mismatch, that is, when the participant selected a portion size image 4 or more images distant from the image corresponding to the presented portion size. For food items presented to the participants with an altered weight compared to the depicted in the image-series, the portion size image closest in weight was considered as its perfect match.

Participants’ estimates of portion sizes were also used to calculate the weight discrepancy in percentage between the chosen portion size image and the portion size presented to the participants. The following formula was used for each of the forty-six foods presented to the participants: [(mean estimated weight (g) – presented portion size (g))/presented portion size (g) × 100]. The weight of the nearest portion size image was used to calculate percent discrepancy for food items presented with altered weight.

Possible differences in portion size estimation accuracy were tested by comparing the mean proportion of correctly classified estimates (correctly classified, as defined above) for all presented food items per participant across sex, level of education (dichotomised into higher (tertiary) education (short, ≤4 years and long, >4 years) and all other), and whether participants had studied food science or nutrition. The non-parametric Mann–Whitney *U* tests were used as the mean proportion of correctly classified estimates per participant were not considered to be normally distributed.

Furthermore, we tested if differences in the food presentation resulted in differences in the accuracy of the portion size estimates. The accuracy of estimates for food items presented with identical *v*. altered weight relative to the portion size image was compared. Also, a similar comparison was made for food items presented as depicted in the image-series *v*. foods presented with a different plate or bowl. Mann–Whitney *U* tests were used comparing the mean proportion of correctly classified estimates made by the participants per food/dish, as data were not considered to be normally distributed.

Statistical analyses were performed using IBM SPSS Statistics for Windows, version 25 (IBM Corp., Armonk, NY, USA). Statistical significance level was set at *P* < 0⋅05.

## Results

The distribution of sex in the sample was relatively balanced (58 % female). The median age was 23 years, ranging from 19 to 44. A majority of the participants (63 %) reported having higher (tertiary) education (short, <4 years or long, ≥4 years). About a quarter of the participants had studied food science or nutrition (median duration: 1¾ years; range: 0⋅5–10; Table 1). All 41 participants completed 46 portion size estimations each, resulting in a total of 1886 comparisons between the presented portion sizes of foods and portion size images.

**Table 1.** Self-reported characteristics of the study participants in the image-series validation study, the Norwegian version of myfood24

Variable	Total (*n* 41)
Sex, *n* (%)	
Female	24 (58 %)
Male	17 (42 %)
Age	
Range	19–44
Median (25 %, 75 %)	23 (21, 27⋅5)
Level of education, *n* (%)	
Upper secondary education	13 (32 %)
Tertiary vocational education	2 (5 %)
Higher (tertiary) education, short^a^	15 (36 %)
Higher (tertiary) education, long^b^	11 (27 %)
Participants having studied food science	
Nutrition, *n* (%)	10 (24 %)
Range, years	0⋅5–10
Median (25 %, 75 %), years	1⋅75 (1, 3⋅5)

a Higher (tertiary) education, short, defined as ≤4 years.

b Higher (tertiary) education, long, defined as >4 years.

**Fig. 3.**
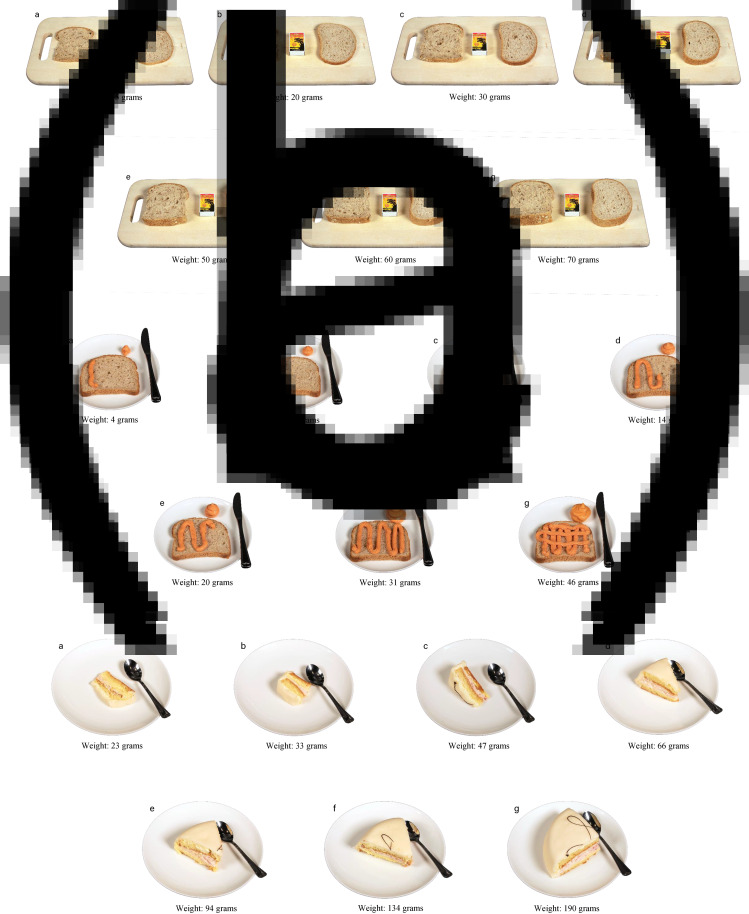
Example of image-series that performed poorly in estimating portion size: (a) bread, (b) caviar spread and (c) marzipan cake.

Across all foods, 55 % (range 7–95 %) of the participants’ portion size estimates were correct, meaning that they matched the portion sizes presented to them with the correct portion size image. Moreover, 38 % of the estimates were matched with the adjacent image, while 6 % were lightly misclassified and 0⋅5 % grossly misclassified by the participants. For thirty-eight of the forty-six presented food items, 90–100 % (mean of 98 %) of estimates were made with the correct or adjacent portion size image. The remaining eight food items were estimated as lightly or grossly misclassified by a mean of 27 % (Table 2 and examples in Fig. 3).

**Table 2.** Proportion of participants’ portion size estimations using the image-series per presented food item in percent classified as correct or adjacent, adjacent, lightly misclassified or grossly misclassified in the image-series validation study, the Norwegian version of myfood24

Food item	Correct or adjacent (%)	Adjacent (%)	Lightly misclassified (%)	Grossly misclassified (%)
Beans	95	27	5	0
Beans^a^	100	5	0	0
Blueberries	98	64	0	2
Blueberries^b^	100	78	0	0
Brownie	95	34	2	2
Brownie^a^	98	37	0	2
Butter (spread)	88	64	12	0
Butter (spread)^a^	81	44	19	0
Candy (without chocolate)	98	69	2	0
Candy (without chocolate)^a^	100	10	0	0
Candy with chocolate	98	13	2	0
Candy with chocolate^a^	98	37	0	2
Carrot cake	100	15	0	0
Carrot cake^a^	100	15	0	0
Caviar (spread)	88	56	12	0
Caviar (spread)^a^	51	27	47	2
Chicken	100	22	0	0
Chicken^a^	100	20	0	0
Jam	100	71	0	0
Jam^b^	76	42	24	0
Liver pâté	88	29	7	5
Liver pâté^a^	100	76	0	0
Marzipan cake	68	58	32	0
Marzipan cake^b^	90	29	10	0
Meat	90	44	7	3
Meat^b^	100	5	0	0
Mexican stew	98	49	2	0
Mexican stew^b^	100	29	0	0
Mexican stew with beans	93	76	7	0
Mexican stew with beans^b^	90	83	10	0
Mexican stew with meat	98	35	2	0
Mexican stew with meat^a^	98	13	2	0
Muesli	100	37	0	0
Muesli^a^	98	83	0	2
Peanuts	100	15	0	0
Peanuts^a^	95	41	5	0
Potato chips	100	37	0	0
Potato chips^b^	100	22	0	0
Raspberries	100	7	0	0
Raspberries^b^	98	37	0	2
Slice of bread	44	29	56	0
Slice of bread^a^	98	35	2	0
Stew (potato based)	95	49	5	0
Stew (potato based)^a^	93	66	7	0
Strawberries	100	12	0	0
Strawberries^b^	98	5	2	0

Correct or adjacent defined as a perfect or partially match, in that participants chose the matching or the closest portion size image corresponding to the portion size of the same food or dish presented to them. Adjacent defined as a partially match, in that participants chose the closest portion size image corresponding to the presented portion size. Lightly misclassified defined as a partially mismatch, in that participants chose an image of a portion size situated 2–3 images distant from the correct portion size image for the presented serving of food. Grossly misclassified defined as a complete mismatch, that is when participants chose a portion size image 4 or more images distant from the image corresponding to the presented portion size.

a Presented portion weight increased by 25 % of the differential to the adjacent portion size image.

b Presented portion weight decreased by 25 % of the differential to the adjacent portion size image.

Table 3 shows the discrepancy in percentage between the portion sizes for foods presented to the participants and the mean of participants’ portion size estimations in gram. Moreover, Table 3 shows the changes in weight made to portion sizes presented with altered weight relative to depicted. The overall mean percent discrepancy between the presented portion sizes and the participants’ portion size estimations was 2⋅5 %, ranging from −33 % for marzipan cake and Mexican stew with beans (presented with decreased weight) to 105 % for caviar, spread (presented with increased weight). Sixteen food items had a percent discrepancy >20 % (Table 3). Food items presented with the weight of the smallest portion size image in an image-series (*n* 6) were all overestimated, with a mean of 43 %. Similarly, all food items presented with the largest portion size image weight (*n* 4) were all underestimated, with a mean of 21 %. The mid-images (images 2–6) had a mean discrepancy <7 % (range: −6 to 4 %), although individual food items show a greater degree of discrepancy (from 50 % for muesli to −33 % marzipan cake).

**Table 3.** Percentage discrepancy between the presented portion size image weight and the mean of participants’ portion size estimations in gram, and the weight alterations for portion sizes presented with altered weight relative to a portion size image in the image-series validation study, the Norwegian version of myfood24

Food item	Presented with identical weight as a portion size image			Presented with altered weight relative to a portion size image			
	Presented portion size (g)	Participants mean estimations (g)	Discrepancy between mean estimation and presented portion size (%)	Presented portion size (g)	Nearest portion size image (alteration) (g)	Participants mean estimations (g)	Discrepancy between mean estimation and nearest portion size image (%)
Beans	98	86	−13	29	25 (+4)	26	3
Blueberries	112	88	−21	68	74 (−6)	55	−25
Brownie	134	121	−10	148	134 (+14)	147	10
Butter (spread)	7	7⋅3	4	5⋅3	5 (+0⋅3)	4⋅9	−2
Candy (without chocolate)	65	45	−31	14	12 (+2)	13	8
Candy with chocolate	78	77	−1	18	15 (+3)	22	47
Carrot cake	47	45	−4	104	94 (+10)	98	4
Caviar (spread)	14	17	21	4⋅5	4 (+0⋅5)	8⋅2	105
Chicken	68	67	−1	77	68 (+9)	72	5
Jam	10	8⋅6	−14	27	29 (−2)	23	−21
Liver pâté	60	50	−17	11	10 (+1)	7⋅9	−21
Marzipan cake	94	63	−33	124	134 (−10)	115	−14
Meat	30	42	41	208	227 (−19)	223	−2
Mexican stew	690	567	−18	73	80 (−7)	85	6
Mexican stew with beans	461	325	−30	633	690 (−57)	460	−33
Mexican stew with meat	80	79	−2	58	50 (+8)	57	15
Muesli	25	27	7	202	174 (+28)	261	50
Peanuts	9	10	11	6	5 (+1)	7⋅2	44
Potato chips	14	19	34	44	50 (−6)	57	13
Raspberries	74	75	2	230	251 (−21)	215	−14
Slice of bread	50	63	25	33	30 (+3)	28	−6
Stew (potato based)	352	327	−7	666	621 (+45)	547	−12
Strawberries	97	104	8	33	36 (−3)	37	2

Each food item was presented twice; once with identical weight relative to a portion size image, and once with portion size weight altered by ±25 % of the differential to an adjacent portion size image.Percentage discrepancy between the mean of participants’ portion size estimations and the presented portion size images calculated as [(mean estimated weight (g) – presented portion size (g))/presented portion size (g)  × 100] (food items presented with altered weight used the nearest portion size image to calculate percent discrepancy).

Portion size estimation accuracy differed across the sexes. Table 4 shows that female participants (*n* 24, median: 0⋅60) chose the correct portion size image more often than male participants (*n* 17, median: 0⋅52; *P* = 0⋅019). No statistically significant difference was observed either for participants with higher (tertiary) education (*n* 26, median: 0⋅57) or other education (*n* 15, median: 0⋅54) in choosing the correct portion size image (*P* = 0⋅613), nor for those that had studied food science or nutrition (*n* 10, median: 0⋅58) compared to those not having studied food science or nutrition (*n* 31, median: 0⋅54; *P* = 0⋅122) (Table 4).

**Table 4.** Comparison of portion size estimation accuracy across participant characteristics in the image-series validation study, the Norwegian version of myfood24

Difference in portion size estimations	Correct estimation		*P*
	Median	IQR	
Sex			
Female (*n* 24)	0⋅60	0⋅11	0⋅019*
Male (*n* 17)	0⋅52	0⋅09	
Level of education			
Higher (tertiary) education^a^ (*n* 26)	0⋅57	0⋅14	0⋅613
Other education (*n* 15)	0⋅54	0⋅13	
Studied food science or nutrition			
No (*n* 31)	0⋅54	0⋅13	0⋅122
Yes (*n* 10)	0⋅58	0⋅06	

Mann–Whitney *U* test. Correct estimation referring to the mean proportion of correctly classified portion size image estimates by the participants for the forty-six presented food items, correctly classified defined as a perfect match between the portion size image chosen by the participant and the portion size of that same food or dish presented to the participant. Median represents the central tendency of the participants mean correct estimates, with interquartile range (IQR) representing the measure of variability.

a Higher (tertiary) education defined as short, ≤4 years or long, >4 years.

* *P* < 0⋅05.

The difference in portion size estimation accuracy per food item showed no statistically significant difference in choosing the correct portion size image for foods presented as depicted (*n* 36, median: 0⋅60) compared to those presented with a different plate or bowl (*n* 10, median: 0⋅63; *P* = 0⋅416), nor for food items presented with identical weight relative to a portion size image (*n* 23, median: 0⋅59) compared to those presented with an altered weight relative to depicted (*n* 23, median: 0⋅61; *P* = 0⋅597) (Table 5).

**Table 5.** Comparison of portion size estimation accuracy across the type of food presentation in the image-series validation study, the Norwegian version of myfood24

Difference in portion size estimations	Correct estimation		*P*
	Median	IQR	
Food presentation			
As depicted (*n* 36)	0⋅60	0⋅51	0⋅416
Not as depicted^a^ (*n* 10)	0⋅63	0⋅43	
Presented weight			
Identical weight (*n* 23)	0⋅59	0⋅49	0⋅597
Altered weight^b^ (*n* 23)	0⋅61	0⋅59	

Mann–Whitney *U* test. Correct estimation referring to the participants’ mean proportion of correctly classified portion size image estimates per dish/food item, correctly classified defined as a perfect match between the portion size image chosen by the participant and the portion size of that same food or dish presented to the participant. Median represents the central tendency of the mean correct estimates per dish/food item, with interquartile range (IQR) representing the measure of variability.

a Presented with a different plate or bowl than depicted.

b Presented with ±25 % of the differential to an adjacent portion size image.

## Discussion

We have recently adapted the British myfood24 for Norway, including the image-series for portion size estimations. During this process, we developed twenty-three image-series, each containing seven portion size images, for typical and frequently eaten Norwegian foods. The validity of the image-series was assessed through a comparison of pre-weighed portions of food to portion size images in the image-series in real time. We observed that most of the portion size estimates were satisfactory, as either the correct or adjacent portion size image was chosen by the participants. Overall, the mean weight discrepancy shows an overestimation of 2⋅5 % between the reported and correct portion size image (ranging from −33 to 105 %). Female participants estimated the correct portion size more often than male participants. The image-series developed for bread, caviar spread and marzipan cake performed poorly. All newly developed image-series, except bread, are included to aid portion size estimation in the Norwegian version of myfood24. New image-series are considered for those that performed poorly.

A few other researchers have published results from validation studies of portion size images that are compared to pre-weighed foods, and subsequently classified as correct, adjacent or misclassified. Findings from three of the studies are in line with the proportion of correct estimates observed in our study^(20,21,32)^. One study, among adults in an African population, reports a higher degree of correct estimates compared to our study^(22)^, while estimates with the correct or adjacent portion size image in our study show similar results to what other have found (ranging from 70 to 95 % of estimates with either the correct or adjacent portion size image^(21,32–34)^). A Danish study found a somewhat lower accuracy when assessing self-served portion sizes in adults and children compared to our study, estimating pre-weighed foods^(35)^.

The flat-slope phenomenon, in which small portion sizes tend to be overestimated and large portions underestimated, was observed in the present study, which is in line with what others have found^(18,21,22,36)^. While the smallest portion sizes were overestimated and the largest portion sizes were underestimated, the remaining portion sizes (representing images 2–6) were on average underestimated by 2 %. The large degree of misestimations for the smallest and largest portion sizes may partly be attributed to the fact that there is only one possible direction for misestimation, compared to the remaining five mid-images with both smaller and larger adjacent images, allowing both over- and underestimation. The five mid-images show an overall tendency to be underestimated, rather than overestimated, although this is not true for all the foods presented. This implies that the mid-images provide an acceptable accuracy at a group level, but that they should be interpreted with caution at an individual level.

Despite the tendency of underestimation observed for the mid-images, an overall overestimation of 2⋅5 % was observed in our study, which is similar to Hernández *et al.*^(16)^ Compared to Vereecken *et al.*, who found an overall underestimation of 15 % when adolescents assessed pre-weighed foods^(33)^, our results show a more accurate overall estimation, which may be explained by our study sample being older^(37)^. This is further supported by the study by Lillegaard *et al.* including children and adolescent showing a wider range in both under- and overestimation (0 to 142 %), compared to our results^(34)^. Other studies validating food images in adults have found both greater and lesser degree of misestimation compared to our results^(20,38)^.

In the present study, we observed a statistically significant difference in choosing the correct portion size image for sex, but not for the level of education or whether they had studied food science or nutrition. Both Ovaskinen *et al.* and Nelson *et al.* found that male participants underestimated portion sizes compared to female participants^(18,20)^, while Naska *et al.* and Venter *et al.* found no significant difference for sex^(21,22)^. We did not observe any difference in the accuracy of portion size estimations for level of education (higher education (short, ≤4 years and long, >4 years) and all other), which corroborate findings from other studies^(20,21)^. We speculate that this may reflect that although the level of education is associated with knowledge and skills, the task of estimating food intake is not a skill taught through the educational system.

The image-series developed for bread performed poorly, similar to a previous study using natural size printed photographs of bread^(20)^. One possible explanation for this result is that we used weight increments fixed in grams rather than percentage to include the Norwegian standard serving sizes for a thin, medium and thick piece of bread^(28)^. Using increments in gram rather than percentage makes distinguishing the difference between portion sizes challenging, as an increase from 20 to 30 g (150 % increase) is visually easier to detect than 60 to 70 g (116 % increase). Further, the image-series depicted two pieces of bread (to illustrate the same weight for different types of bread) placed on a wooden cutting board with a matchbox as a reference measure (Fig. 3). It is difficult to conclude what caused the poor performance, as the image-series differed from the others in multiple ways. Participants expressed difficulty in applying the image-series, as the two depicted pieces of bread were presented separately during the validation study.

Spreads represent six of the eight food items most often misclassified in our study, specifically: jam, liver-pâté, butter and caviar (Table 2). Other studies have also reported poor estimates^(20)^ and high percentage of error^(22)^ when assessing portion sizes of spreads. Image-series of spreads were depicted on a piece of bread with an equal amount alongside to illustrate the quantity. Caviar was depicted as squeezed out of the tube packaging (Fig. 3). Spreads had the smallest portion sizes and lowest percent weight increment relative to other image-series developed in this study. During the validation study, spreads were only presented as spread on a piece of bread (caviar included). The small weight increments used for spreads could explain the degree of misestimation. For butter, the three smallest portion size images are 3, 4 and 5 g, respectively. The differences are visually noticeable in the image-series, but in a real-time setting, without the amount of spread illustrated alongside, it may be difficult to estimate. Additionally, estimating spreads on a piece of bread is challenging to quantify compared to more tangible food items, such as pieces of candy. Some participants expressed difficulties estimating portions of spreads, as it was unclear whether they should consider both the spread depicted on the piece of bread and the amount alongside.

The image-series for marzipan cake also performed poorly, similar to other findings using digital pictures of pies to assess portion size^(21)^. The three smallest images in our series differed from the remaining four (Fig. 3), in that the small portions were depicted lying on the side, while the larger portions were depicted as upright triangular pieces.

In the present study, bread, some spreads and marzipan cake performed poorly. What foods are most critical to assess accurately in dietary assessment will always depend on the research question of interest. Bread, and subsequently spreads, are frequently consumed in Norway^(24)^, emphasising the importance of developing accurate tools to estimate portion sizes for these foods.

### Strengths and limitations

A strength in the present validation study is the use of pre-weight foods as the reference tool, which is in line with Amoutzopoulos *et al.* recommendations for validating portion size estimation tools^(7)^.

Evaluating the validity of image-series is in several other studies conducted using the perception or conceptualisation and memory approach^(39)^. This study evaluated the newly developed image-series using the perception approach. The advantages of using this approach are that it excludes participant biases related to memory and recall and provides direct feedback on the image-series applicability to estimate portion sizes. Furthermore, as the participants were not assessing their own diet, but rather a selection of random portion sizes presented to them, one could hypothesise that this reduces the degree of social desirability.

We excluded individuals having recorded their diet during the last year, based on the assumption that people who had registered their diet would estimate portion size more accurately than the general public. Hence, we argue that there is no reason to believe that our results are better than in the general population. Moreover, our study had a relatively even distribution of sex and a fair representation of the age group (58 % female and age range of 19–44 years, respectively). Yet, the sample size and education level of participants limits the generalizability. Recruitment at the university can explain why a majority of the participants had higher (tertiary) education. Additionally, the proportion of highly educated participants may be under-reported, as an unclear phrasing of the said question may have caused participants to select a lower level of education. Using the perception approach to evaluate the image-series in this study may limit the generalizability of the results to a situation relying on participants memory and conceptualisation for dietary assessment. A potential study limitation is the unnatural setting in a large university kitchen, with other participants working their way through the presented food items. This may not reflect the same results as estimating portion sizes individually in a natural setting.

## Conclusion, implication and further research

The newly developed image-series for traditional and frequently eaten Norwegian foods performed satisfactorily in estimating portions of pre-weighed foods using a perception approach, except for a few food items (bread, caviar spread and marzipan cake). The participants matched more than half of the forty-six presented portion sizes with the correct portion size image, and more than 90 % with either the correct or the adjacent portion size image. Overall, there was an overestimation of 2⋅5 % (ranging from −33 to 105 %). The ‘flat-slope’ phenomenon was observed for the largest and smallest portion sizes, and although the remaining five mid-images in the twenty-three image-series show an overall acceptable accuracy (<7 %), they mask a varying degree of misestimation. All newly developed image-series, except for bread, were included to aid portion size estimation in the Norwegian version of myfood24. This study adds to the importance of validating portion size estimation tools.

The finding that the image-series for bread and spreads performed poorly is of significant importance, as bread is a staple food in the Norwegian diet. The accuracy of portion size estimation in the present study is comparable to what others have found. By conducting this study, it was revealed which of our new image-series need to be modified and re-validated. New image-series are planned for those that performed poorly.

## Supplementary material

For supplementary material accompanying this paper visit https://doi.org/10.1017/jns.2020.58. click here to view supplementary material
