# Supplementary material for: Development and evaluation of image-series for portion size estimation in dietary assessment among adults
Source: J Nutr Sci. 2021 Jan 13;10:e3. doi: 10.1017/jns.2020.58 (PMC8057404; doi:10.1017/jns.2020.58)
Supplement: Supplementary file 1 [file S2048679020000580sup001.zip › Salvesen,_Supplementary_file_1.pdf]

**Supplementary file 1**

Table S1. Photography specifications for the new images-series in the Norwegian version of myfood24

| Equipment | Specifications |
|---|---|
| Photographer | Morten Rosenvinge, University of Agder |
| Camera | Canon EOS7D |
| Optics | Canon 24-70mm ultrasonic |
| Background | Black cotton cloth with crossed horizontal and vertical lines |
| Lighting | Two Litepanels LED-lights (with additional plastic film) |
| Camera angle | 40° |
| Distance | Camera lens to middle of plate: 58 cm |
| Image editor | Adobe Photoshop |
