# Supplementary material for: Development and evaluation of image-series for portion size estimation in dietary assessment among adults
Source: J Nutr Sci. 2021 Jan 13;10:e3. doi: 10.1017/jns.2020.58 (PMC8057404; doi:10.1017/jns.2020.58)

Supplementary file 2

Fig. S1. Photography set-up

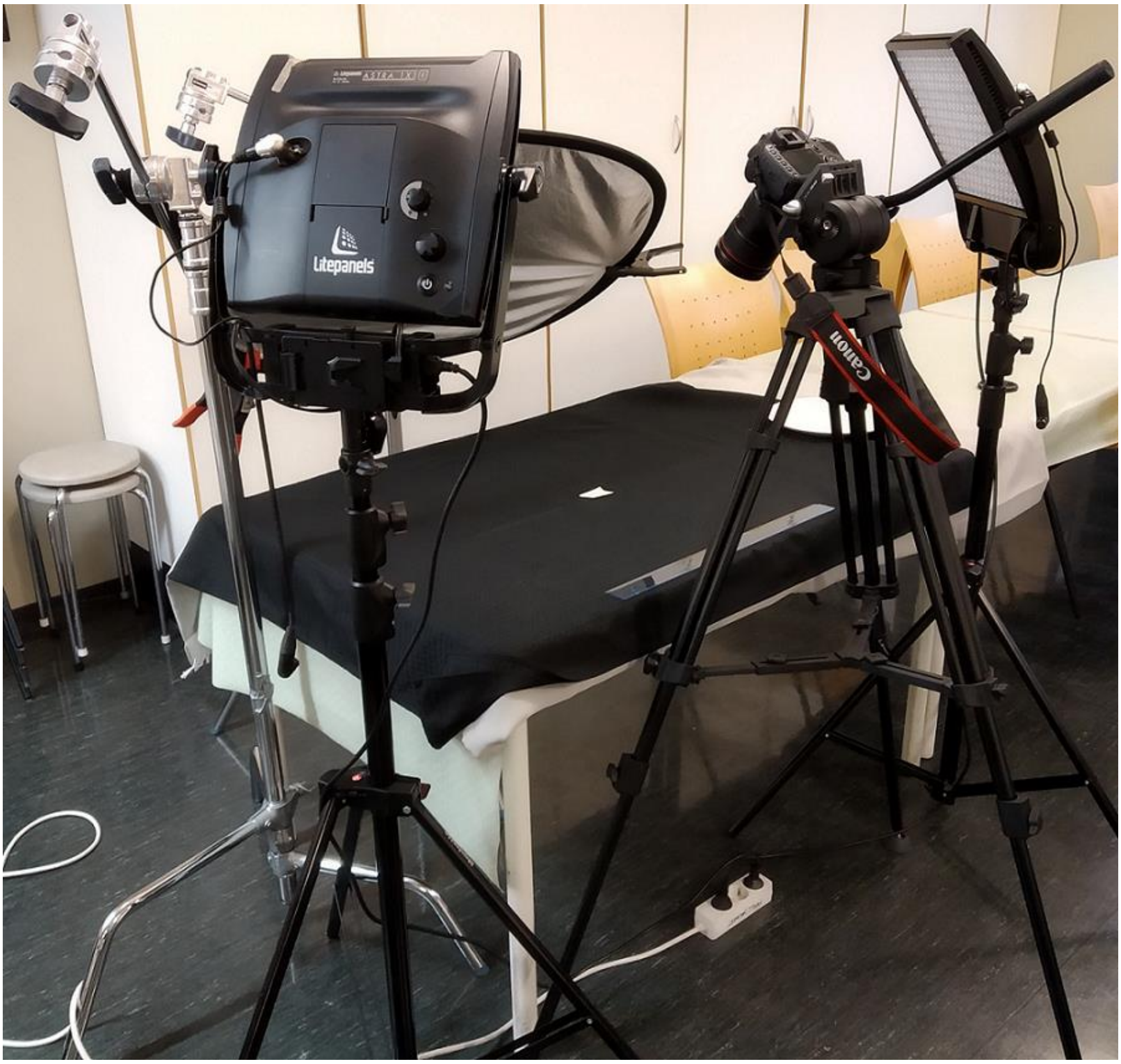

Fig. S2. Example of photographing the image-series for bread

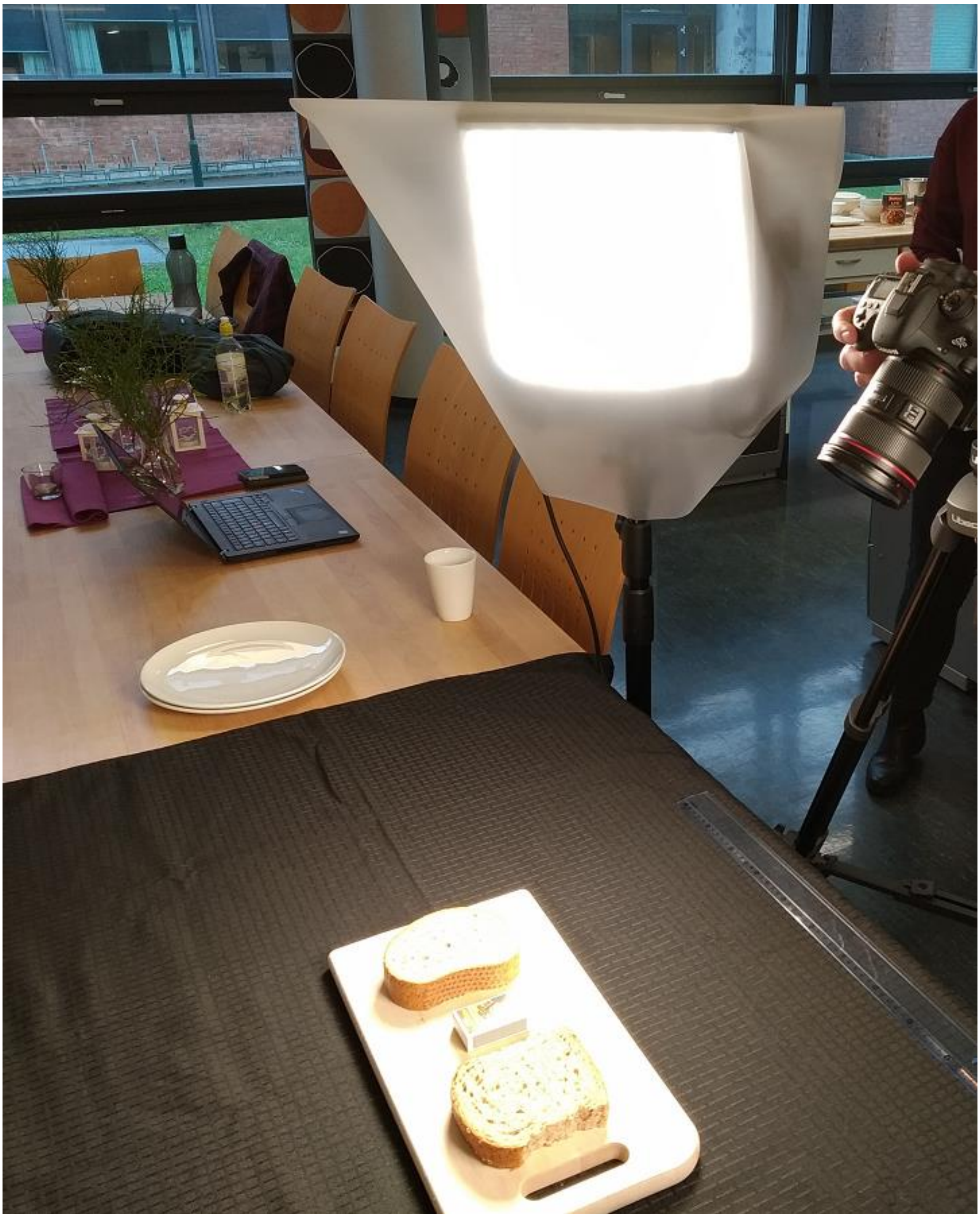

Supplement: Supplementary file 1 [file S2048679020000580sup001.zip › Salvesen,_Supplementary_file_2.pdf]
