# Supplementary material for: Development and evaluation of image-series for portion size estimation in dietary assessment among adults
Source: J Nutr Sci. 2021 Jan 13;10:e3. doi: 10.1017/jns.2020.58 (PMC8057404; doi:10.1017/jns.2020.58)
Supplement: Supplementary file 1 [file S2048679020000580sup001.zip › Salvesen,_Supplementary_file_3.pdf]

Supplementary file 3. Image-series developed but not presented in Salvesen L, Engeset D, Øverby NC and Medin AC “Development and evaluation of image-series for portion size estimation in dietary assessment among adults”

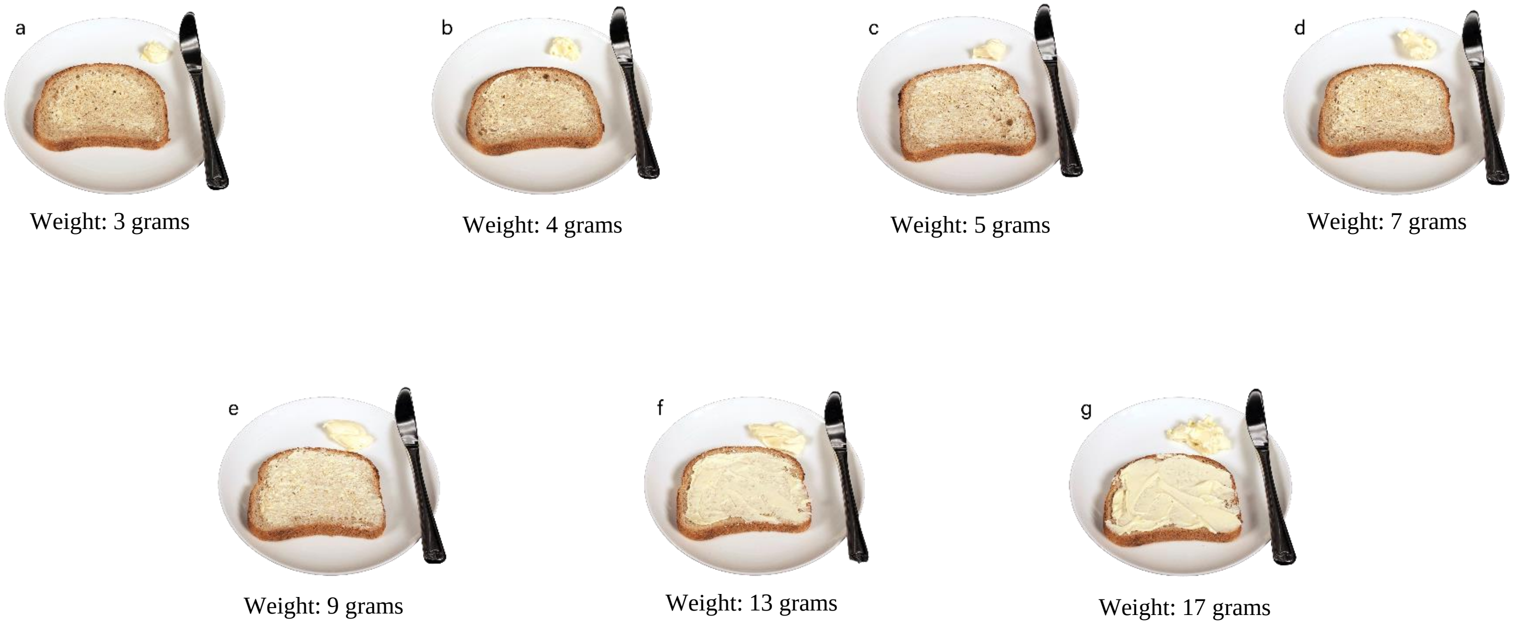

Fig. S1. Image-series for butter

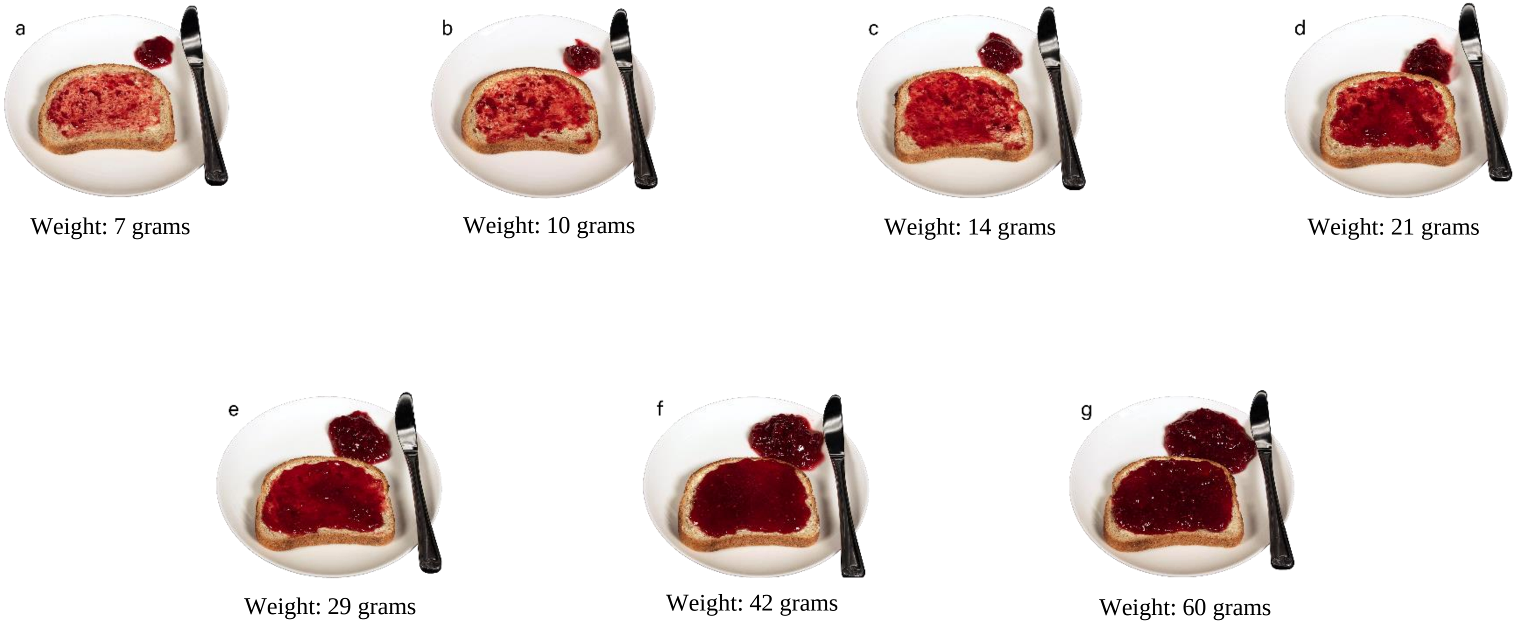

Fig. S2. Image-series for jam

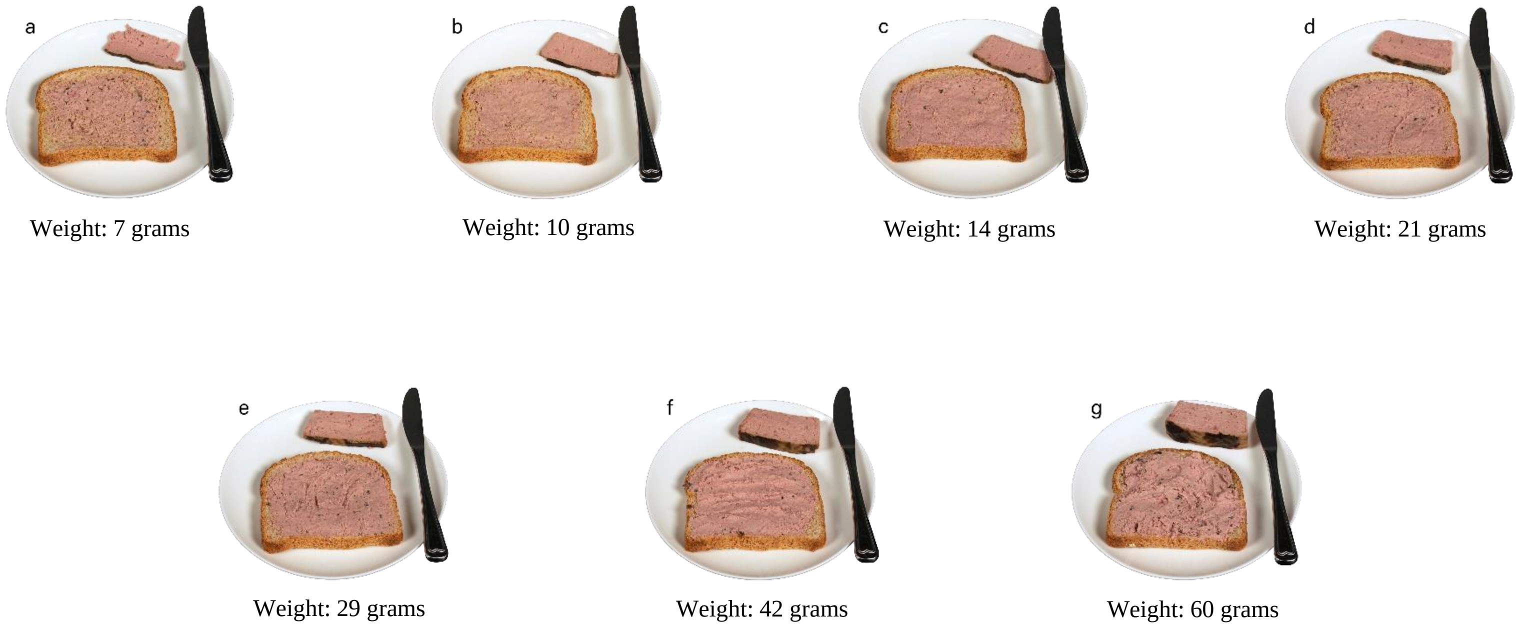

Fig. S3. Image-series for liver-pâté

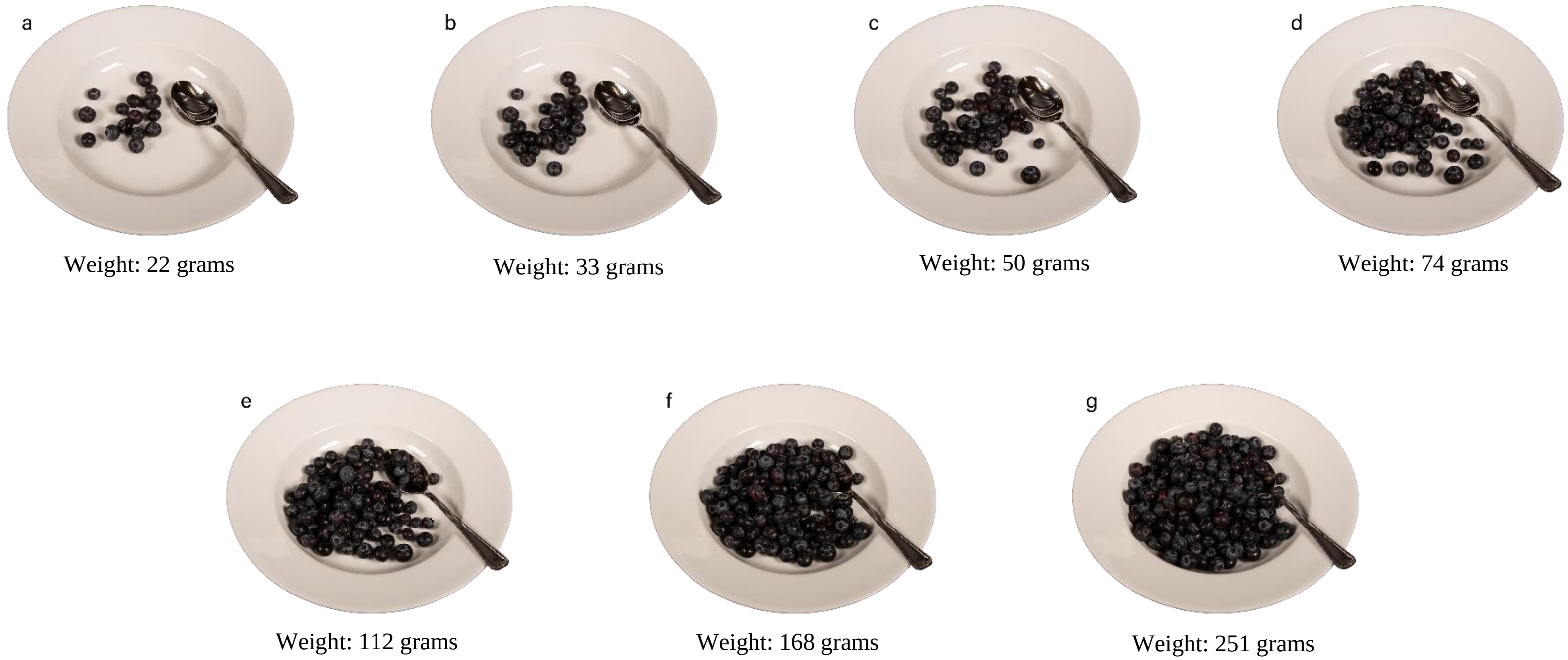

Fig. S4. Image-series for blueberries

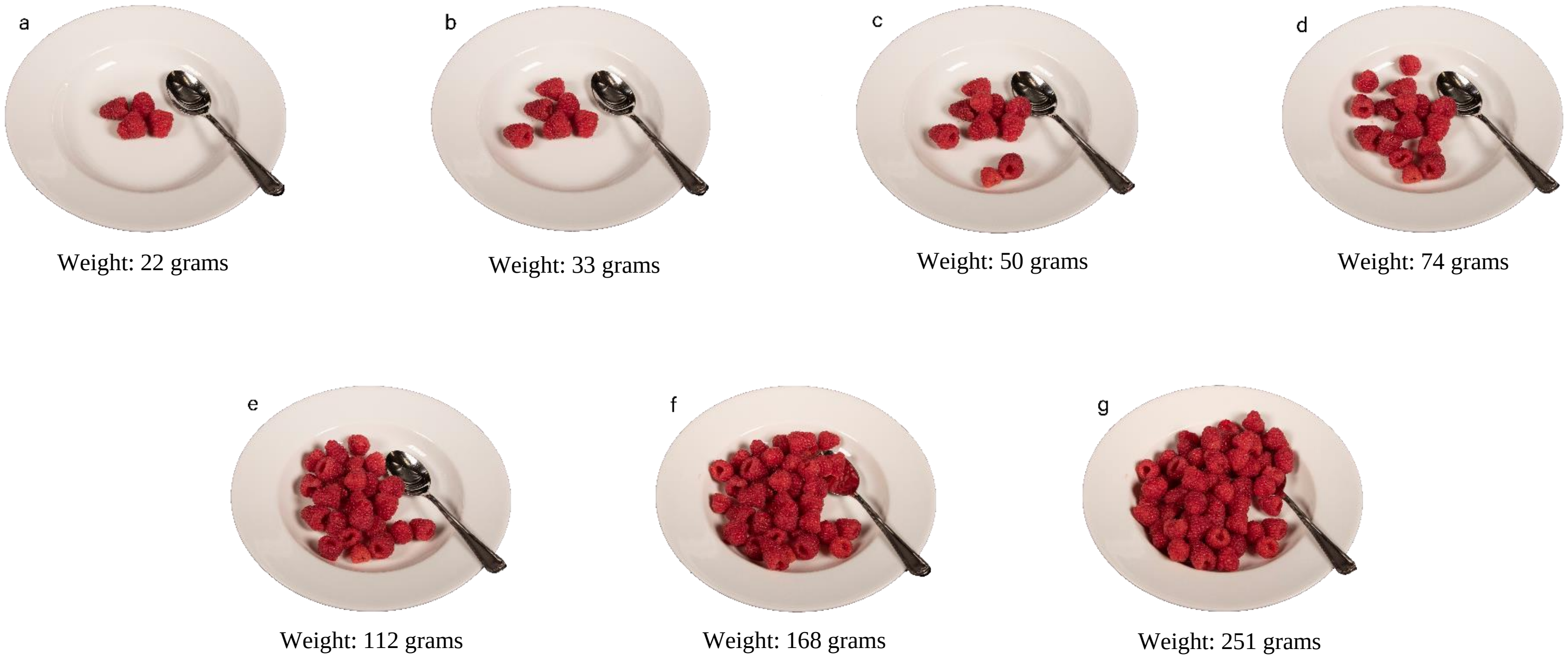

Fig. S5. Image-series for raspberries

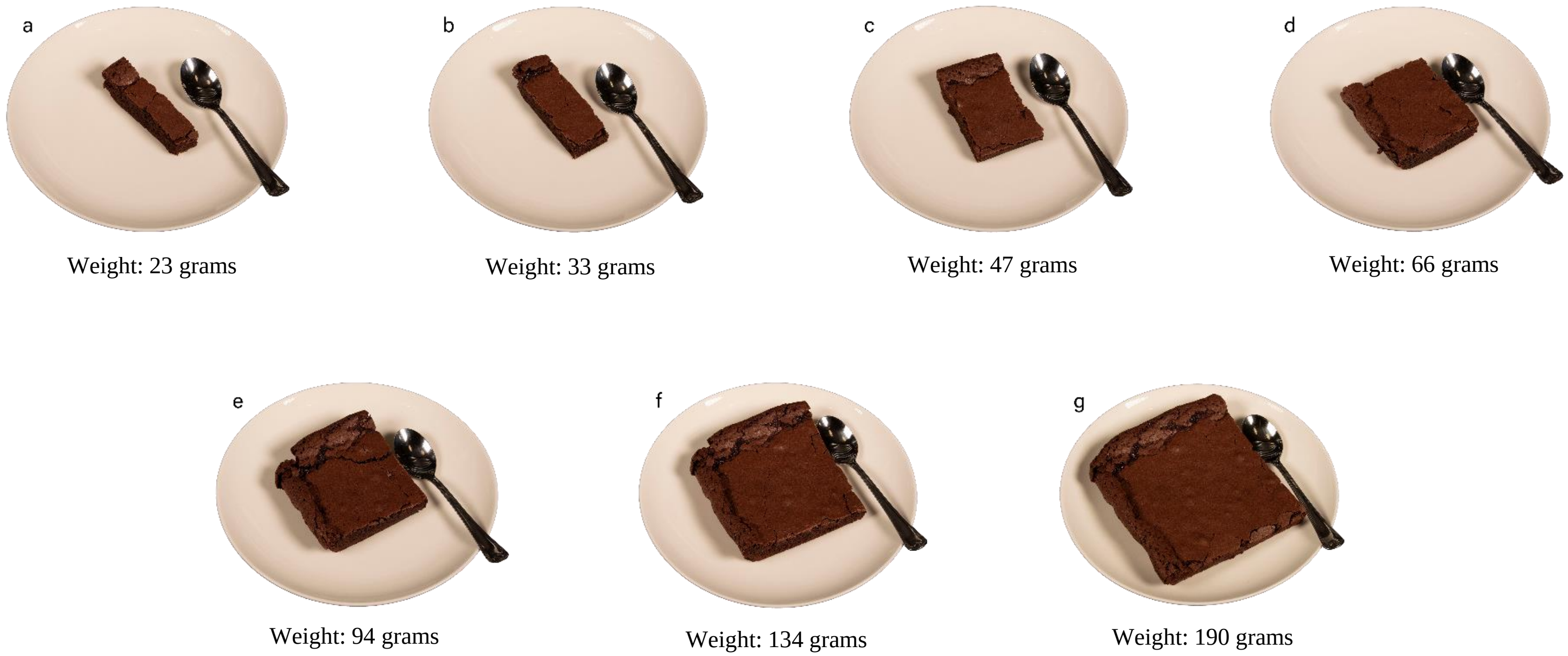

Fig. S6. Image-series for brownie

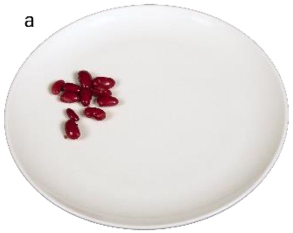

Weight: 16 grams

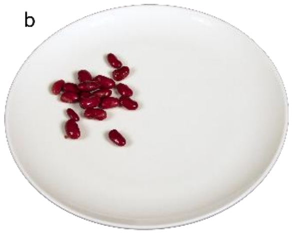

Weight: 25 grams

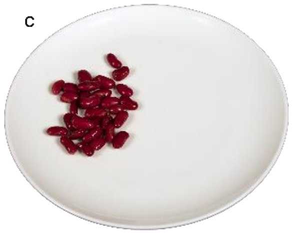

Weight: 40 grams

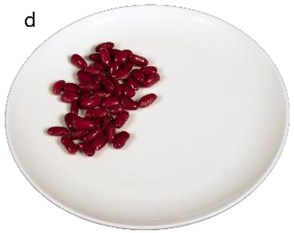

Weight: 62 grams

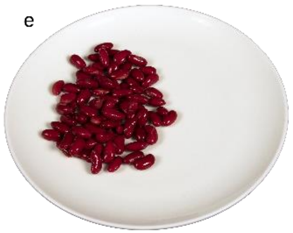

Weight: 98 grams

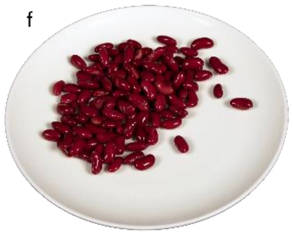

Weight: 153 grams

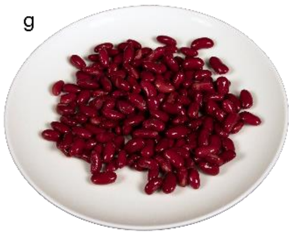

Weight: 240 grams*

Fig. S7. Image-series for beans, kidney, *percent increment 57%; equals one box of canned kidney beans

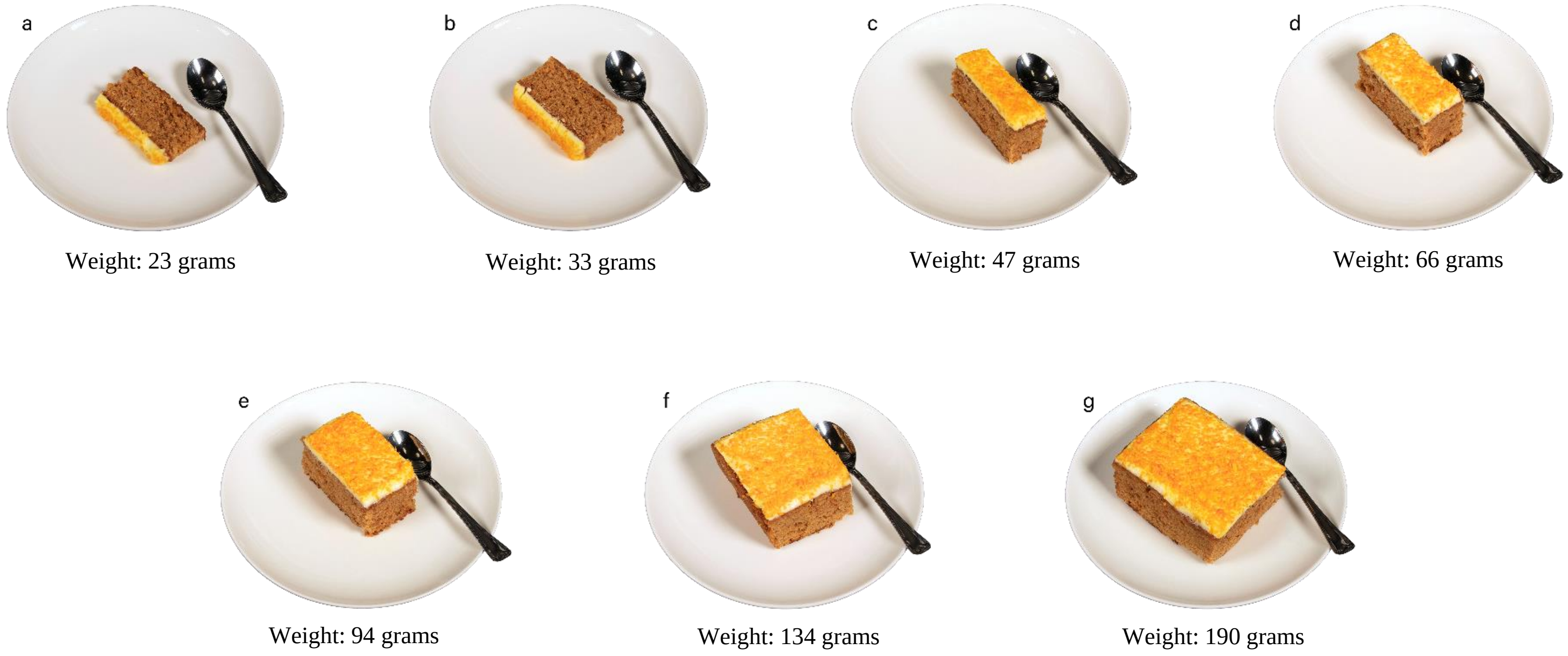

Fig. S8. Image-series for carrot cake

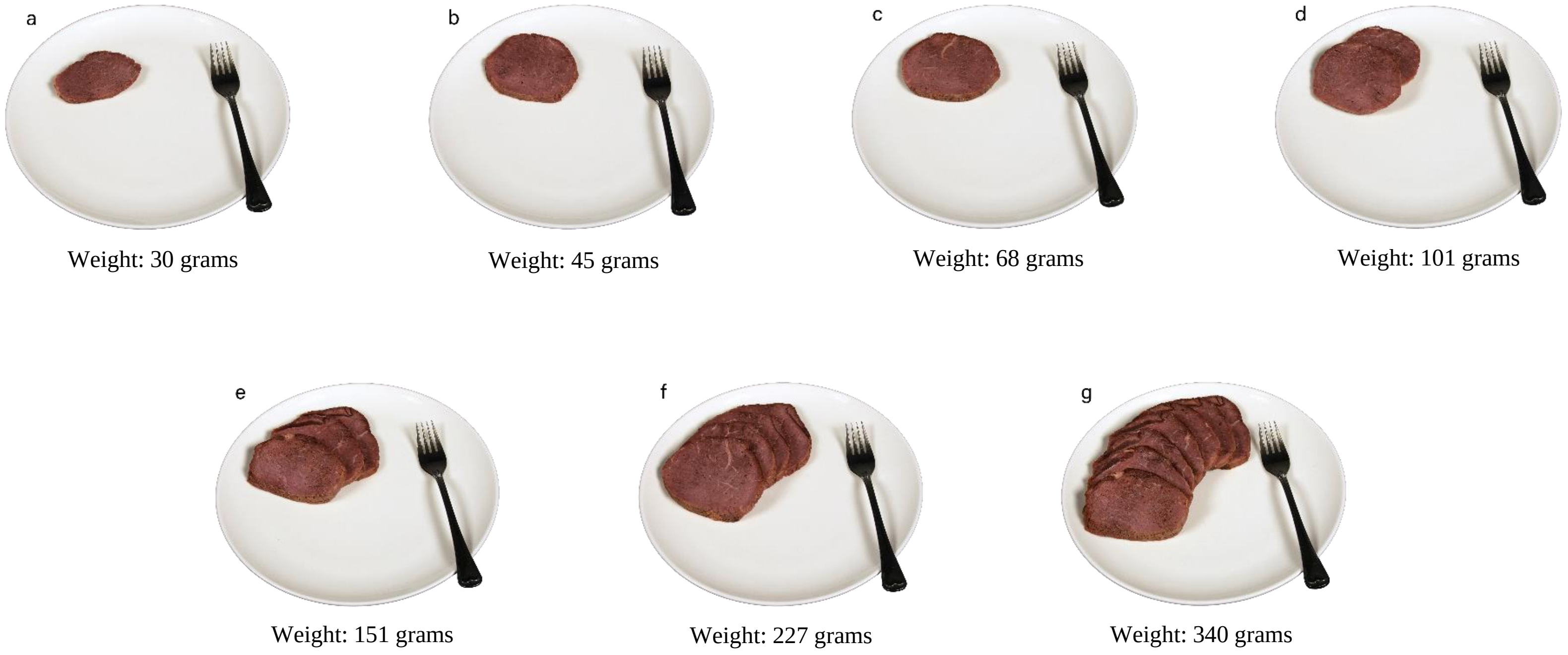

Fig. S9. Image-series for meat

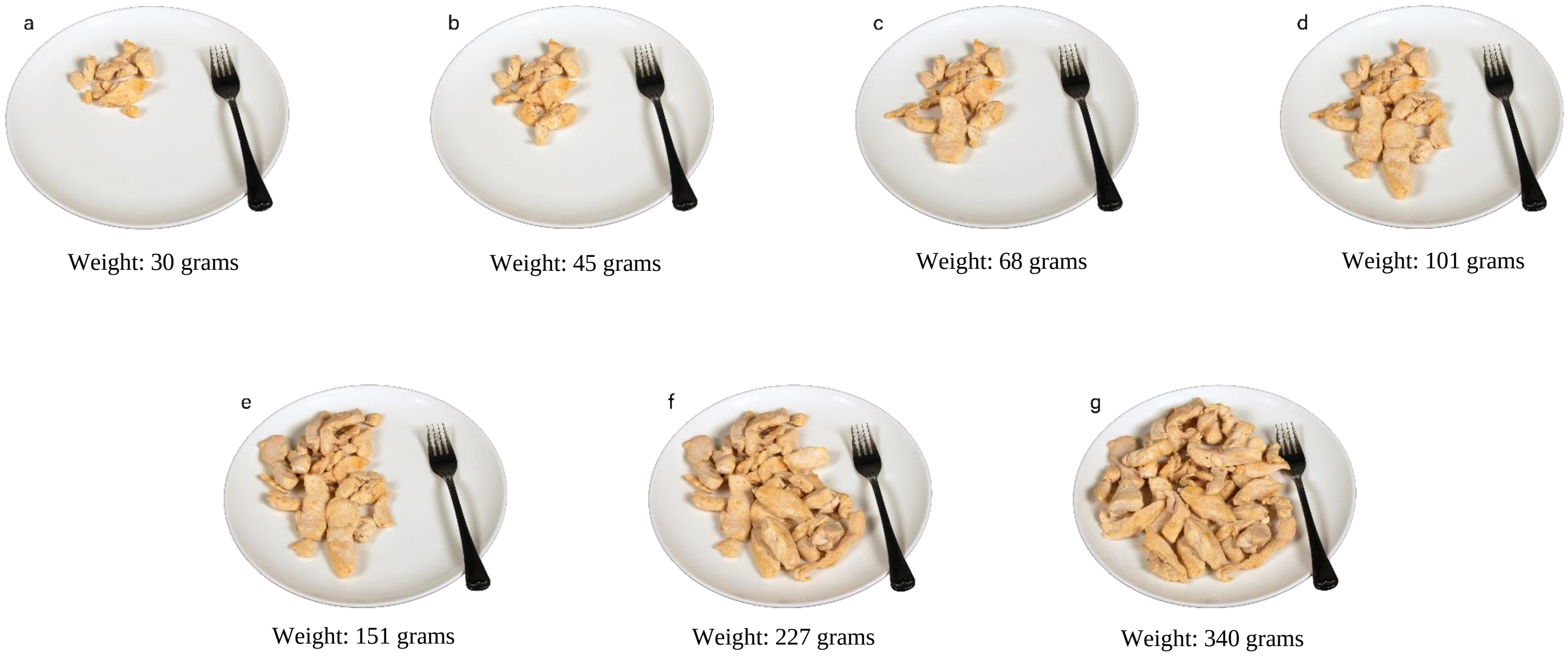

Fig. S10. Image-series for chicken

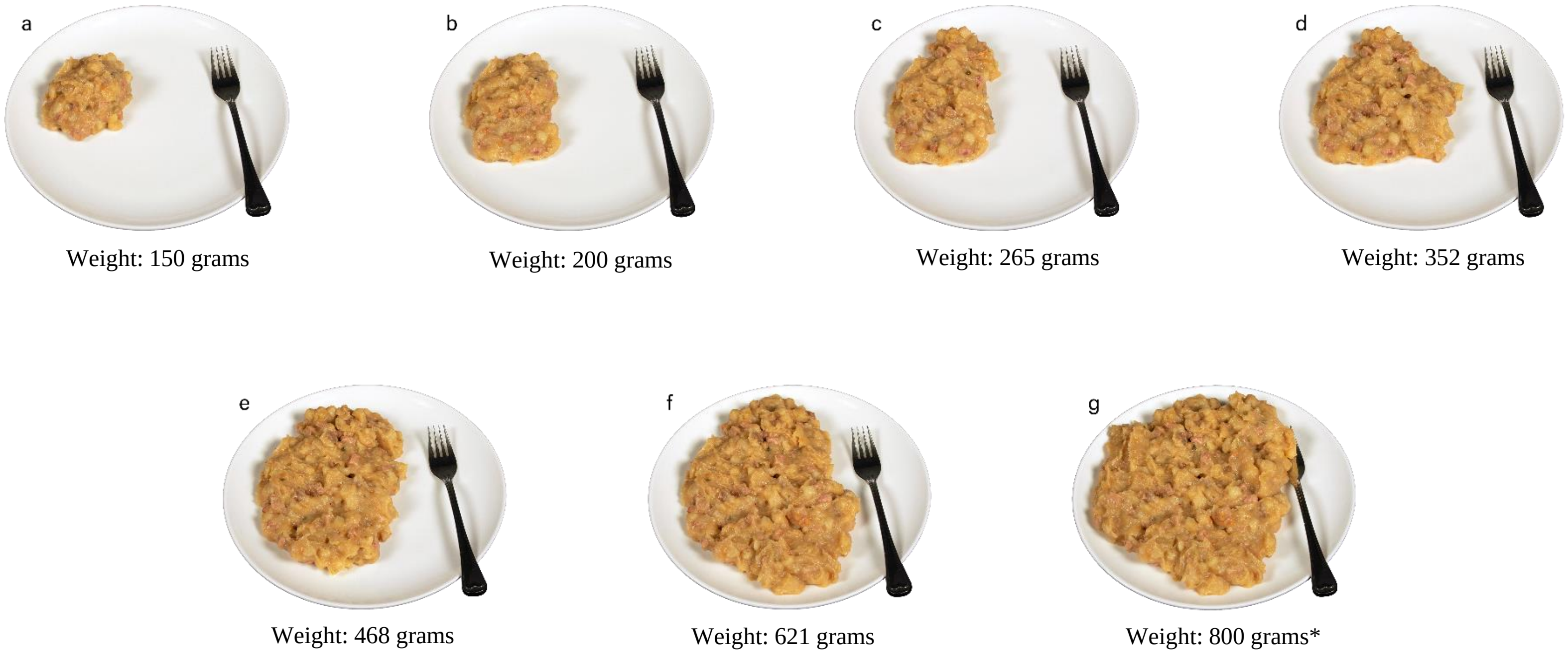

Fig. S11. Image-series for stew (potato based), *percent increment 29%; 800 grams equals one can of stew

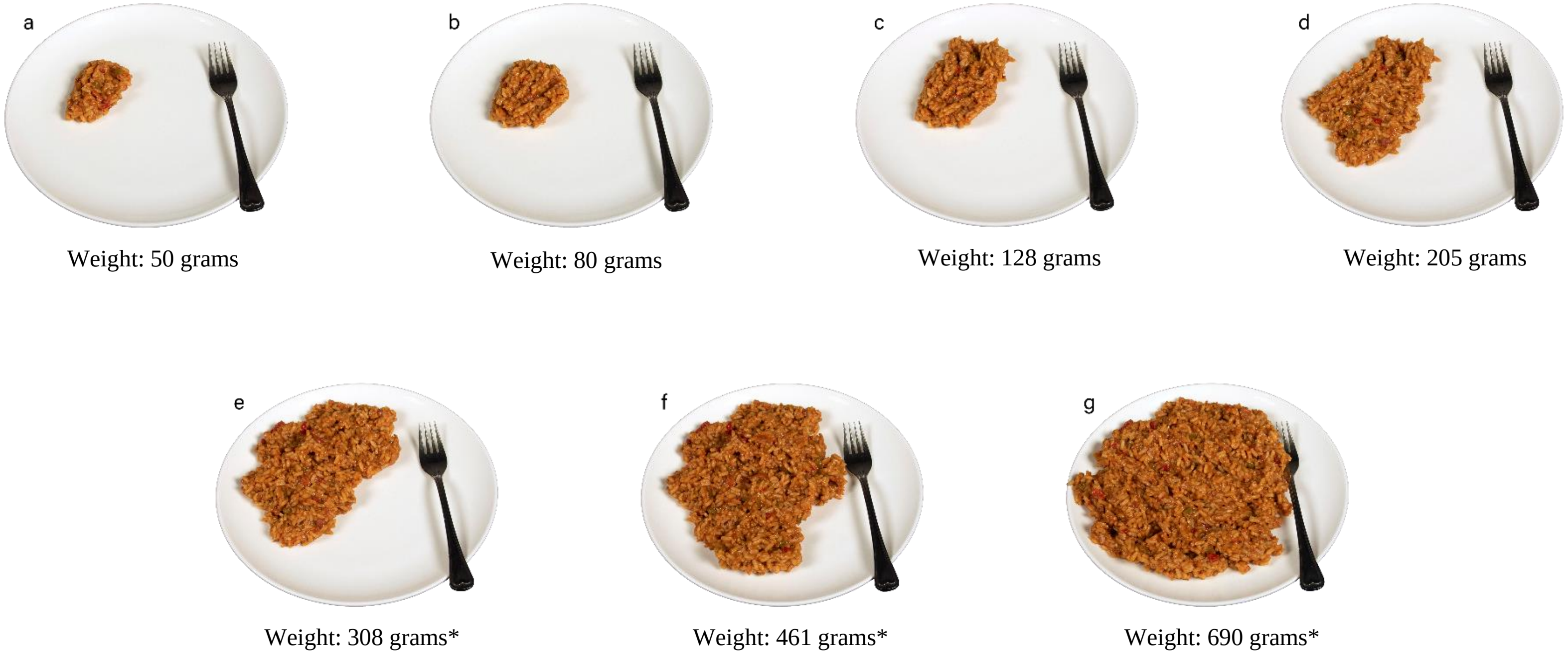

Fig. S12. Image-series for Mexican stew, *percent increment 50%

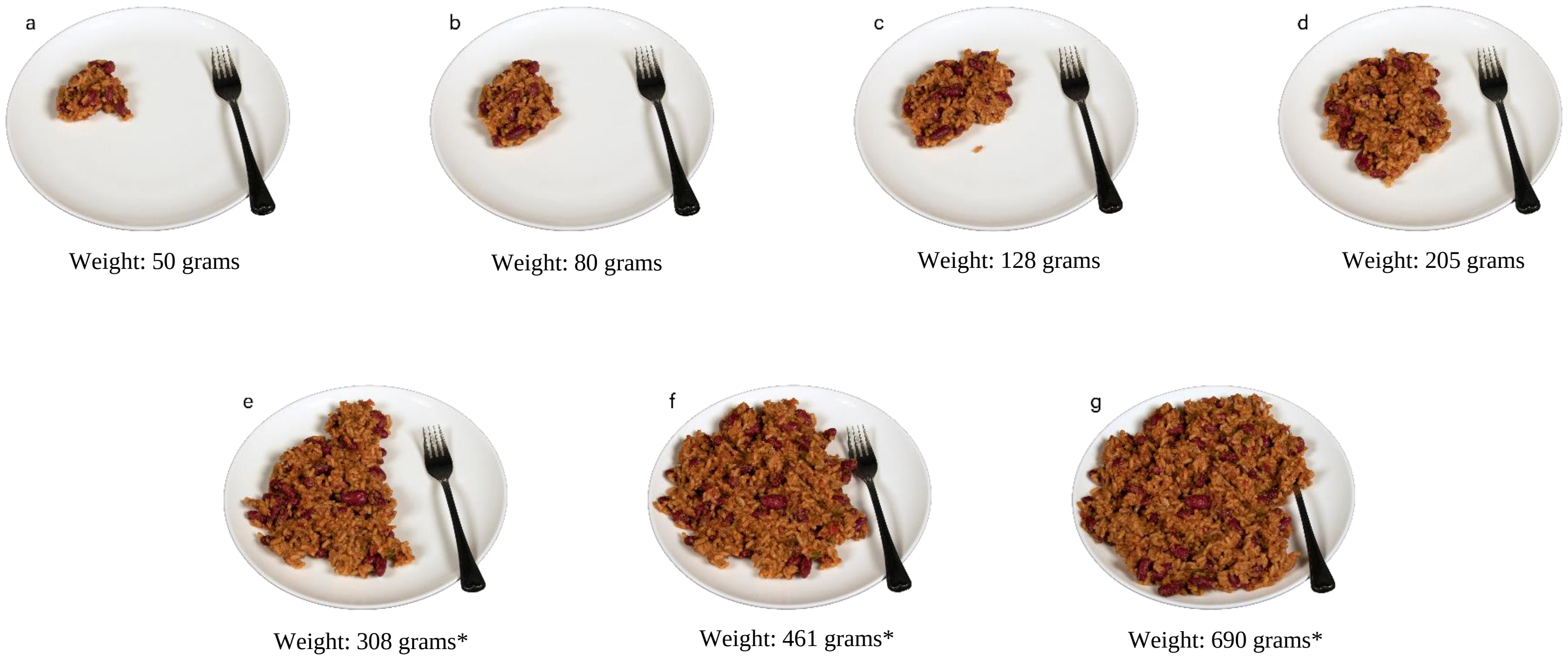

Fig. S13. Image-series for Mexican stew with beans, *percent increment 50%

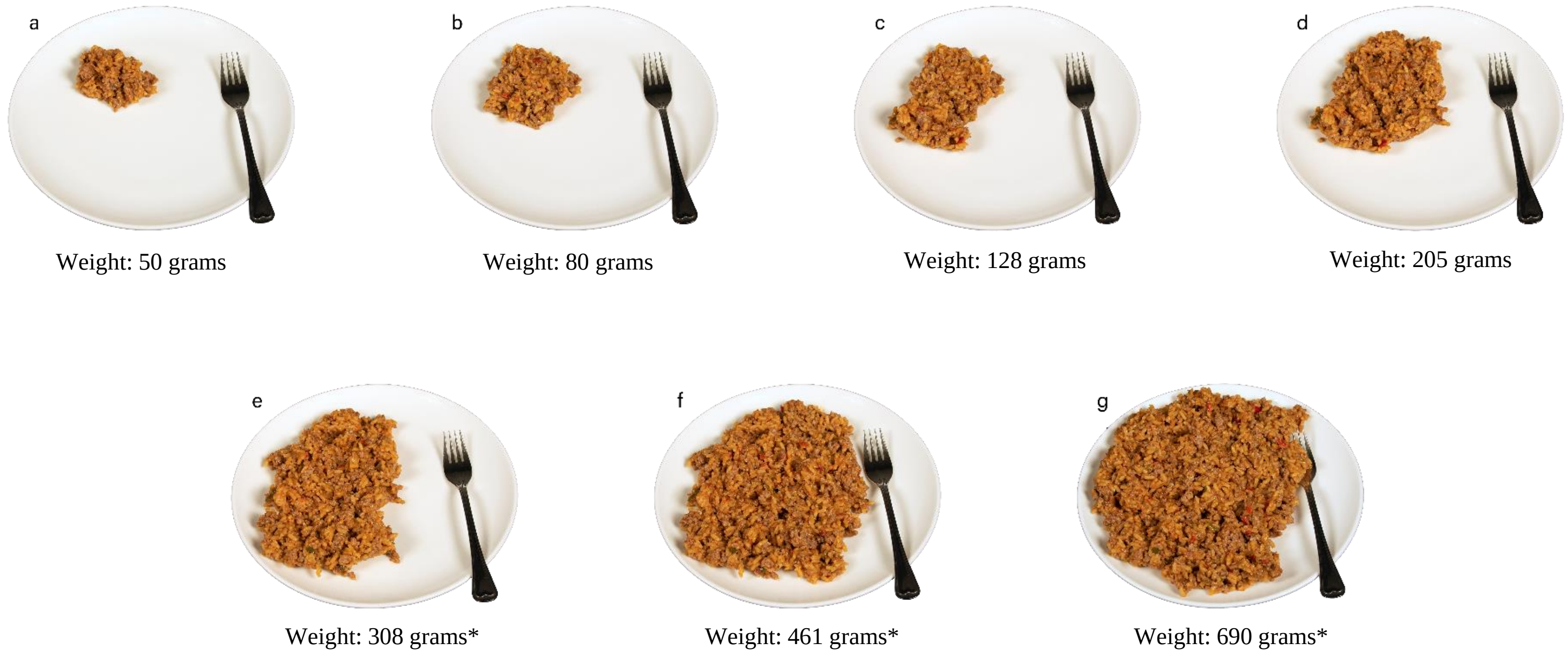

Fig. S14. Image-series for Mexican stew with meat, *percent increment 50%

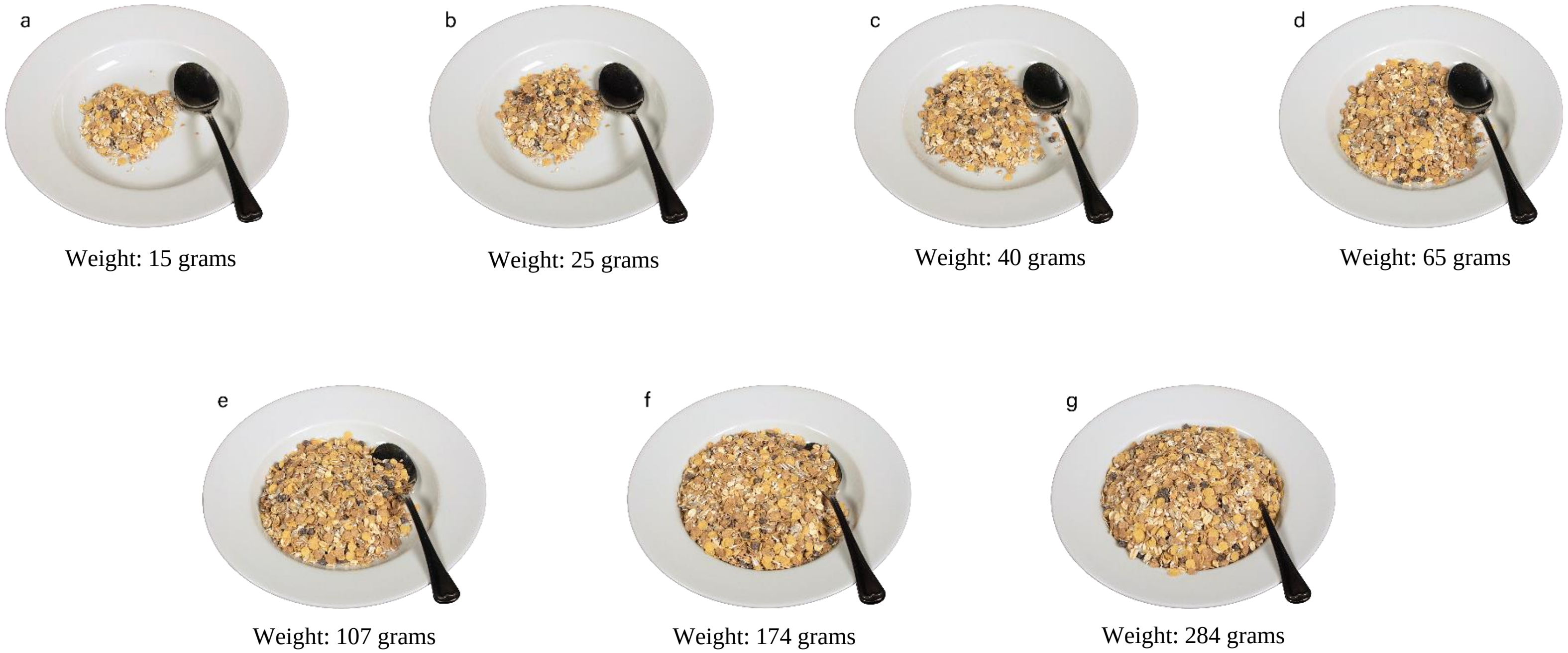

Fig. S15. Image-series for muesli

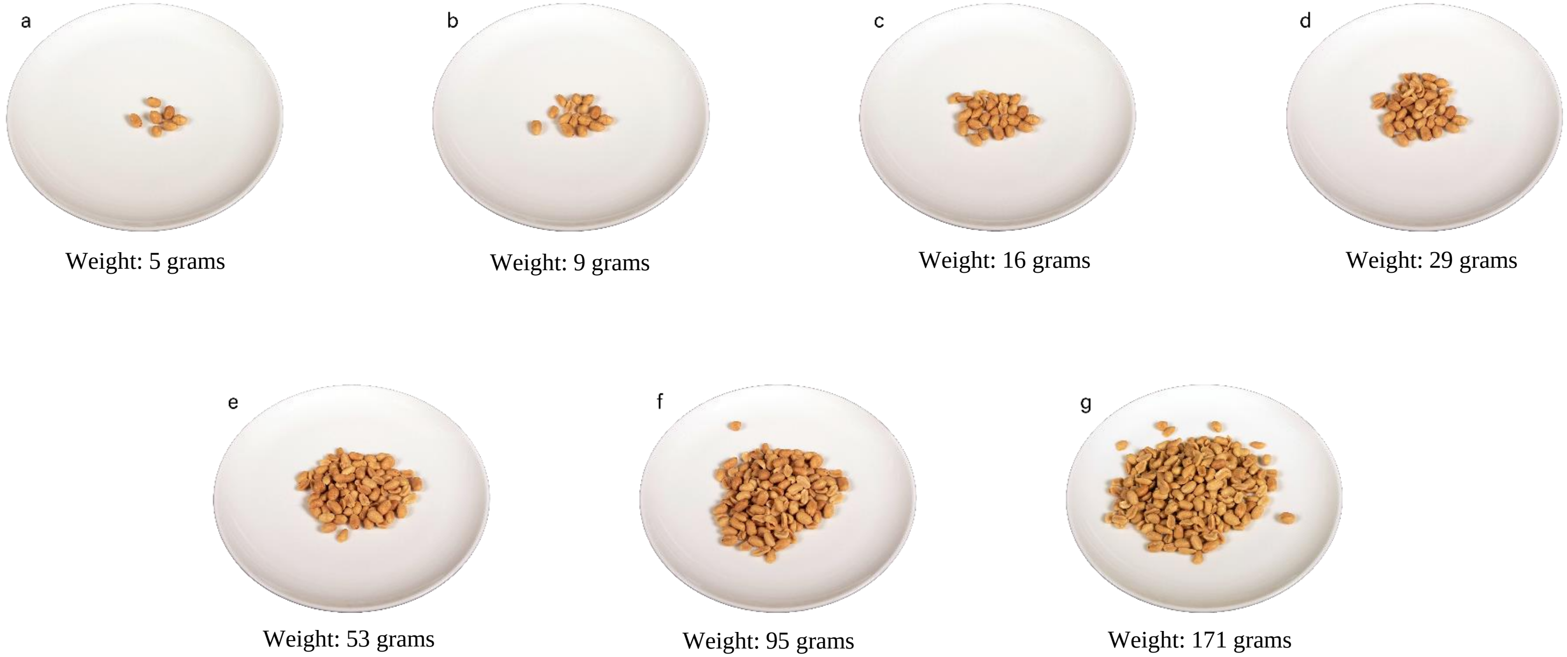

Fig. S16. Image-series for peanuts

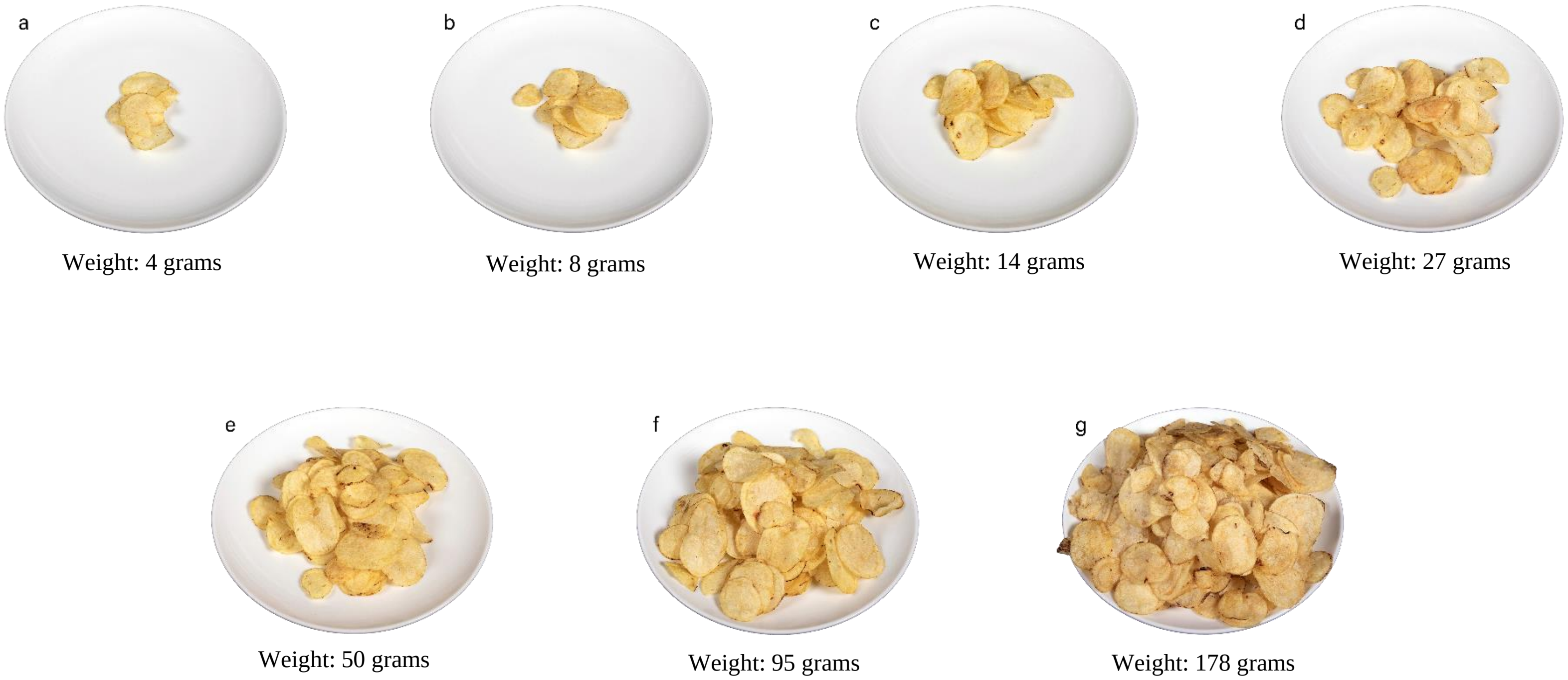

Fig. S17. Image-series for potato chips

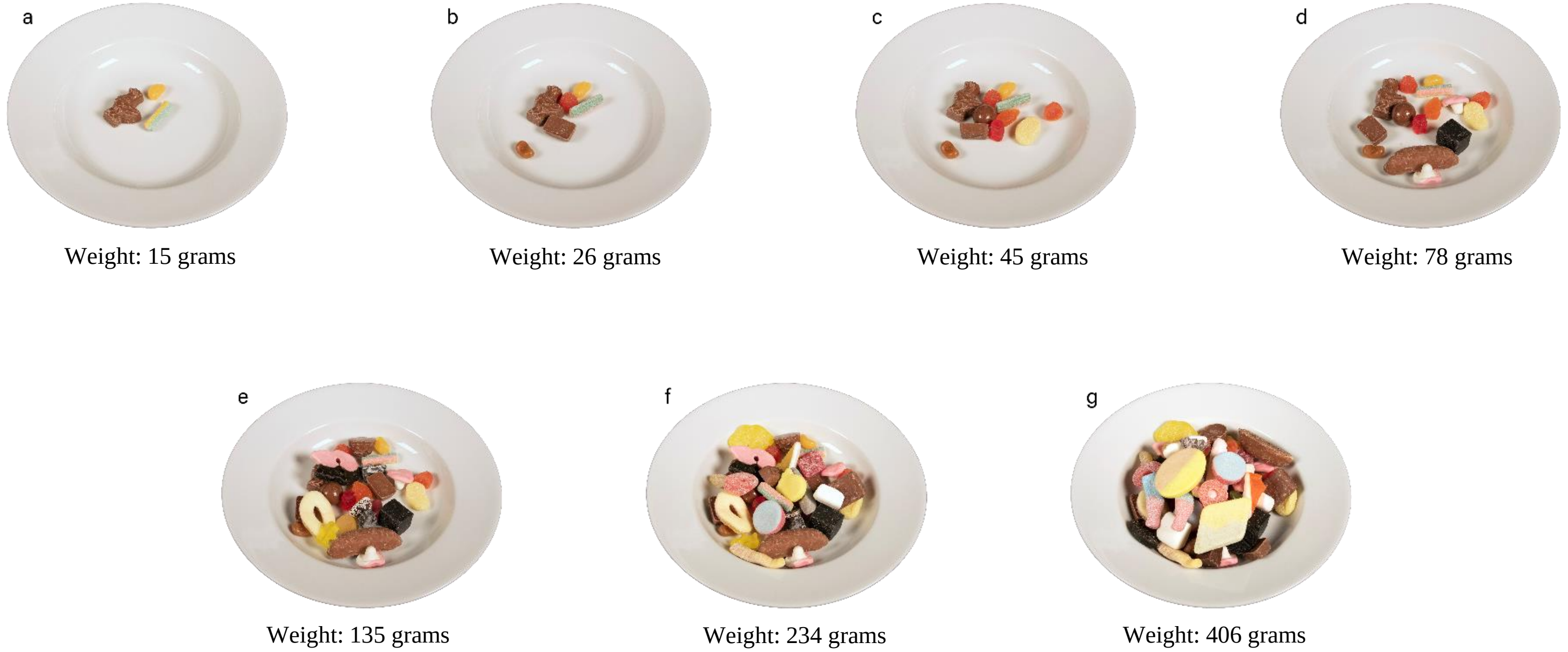

Fig. S18. Image-series for candy with chocolate
